# In silico analysis of the potential effects of the disease-associated point mutations on the kinase activity of TGFBR1 and TGFBR2 receptors

**DOI:** 10.1186/2045-7022-4-S2-P17

**Published:** 2014-03-17

**Authors:** Pierre Rougé, Annick Barre, Jean-Philippe Borges, Stéphanie Caze-Subra, Camille Gironde

**Affiliations:** 1 UMR IRD/UPS 152, Faculté de Pharmacie, 39 chemin des Maraichers, Toulouse, 31062, France; 2 UMR IRD/UPS 152, Biology, Toulouse, France; 3 LISBP Plateforme Biopuces, Toulouse, France

## 

Annick Barre, Jean-Philippe Borges, Stéphanie Caze-Subra, Camille Gironde, Pierre Rougé Université de Toulouse; UPS; UMR 152 Pharma-Dev; Université Toulouse 3, Faculté des Sciences Pharmaceutiques, F-31062 Toulouse cedex 09, France Institut de Recherche pour le Développement (IRD); UMR 152 Pharma-Dev; F-31062 Toulouse cedex 09, France TGFBR1 and TGFBR2 mutations associated with Marfan syndrome-related diseases have been recently suspected to play a key role in triggering the immune changes responsible for allergic diseases. In this respect, patients suffering from Marfan and Loeys-Dietz syndromes exhibited higher rates of (food) allergies, compared to other allergic patients. A possible common mechanism underlying both disorders should consist of the altered ability of TGBR1 and TGFBR2 receptors to properly respond to the recognition of the TGFβ signal, thus preventing the TGFβ−signaling pathway from operating correctly. To check such a hypothesis, we performed an in silico analysis to predict the effects of point mutations in TGFBR1 and TGFBR2 receptors associated to the Marfan and Loeys-Dietz syndromes, on the kniase activity of the serine-kinase domain of both receptors. The most frequently observed disease-related point mutations in TGFBR1 and TGFBR2 receptors essentially occur at hot spots that are located in the surrounding vicinity of the catalytic loop of the kinase domain. They are susceptible to modify the local conformation of the catalytic loop and thus to alter the phosphorylative activity of the kinase domain for the corresponding substrates, e.g. the SMAD proteins. Additionally, another point mutation associated to the Loeys-Dietz disease should modify the local conformation close to the P glycin-rich loop of the kinase domain, involved in the anchorage of an ATP molecule which serves as a phosphate-group donor necessary for the phosphorylation process of the substrate. This point mutation should also prevent a correct operation for the kinase domain of the TGFβ receptors. Although they remain speculative, our modeling results bring some credit to the hypothesis that selected point mutations occurring at hot spots in both TGFBR1 and TGFBR2 kinase domains, consist of a possible driven mechanism in promoting allergic disorders via an alteration of the phosphorylation capacity of the kinase domain of the TGFβ receptors. Obviously, these predictions need to be substanciated by experimental results.

